# International Medical Collaboration: Lessons from Cuba

**DOI:** 10.3390/children3040020

**Published:** 2016-10-18

**Authors:** Mauro Castelló González, Reinaldo Pons Vásquez, David Rodriguez Bencomo, Imti Choonara

**Affiliations:** 1 Camaguey Children’s Hospital, Camaguey 70100, Cuba; cgmauro.cmw@infomed.sld.cu (M.C.G.); rpons@finlay.cmw.sld.cu (R.P.V.); daruben.cmw@infomed.sld.cu (D.R.B.); 2 Academic Unit of Child Health, The Medical School, University of Nottingham, Derbyshire Children’s Hospital, Derby DE22 3DT, UK

**Keywords:** child health, Cuba, access to healthcare

## Abstract

Over 50,000 Cuban health professionals are currently working overseas in 67 different countries. They work in conjunction with local health professionals. The majority work in primary care in deprived areas. The aim is to reduce morbidity and mortality but also improve health in the long term by training local health professionals, and building both institutions and a structure to deliver health care alongside educating the local population. Cuba is a small, middle-income country. It has, however, made a significant international contribution in relation to medical collaboration. Cuba’s international collaboration is based on the principles of social justice and equity for all. It has set an example for other countries to emulate.

## 1. Introduction

Cuba currently has over 50,000 health professionals working in 67 different countries [1,2]. This is a greater number of health professionals than Médecins Sans Frontières (MSF), The Red Cross and Unicef combined [3]. The majority work in 25 different Caribbean and Latin American countries. Cuba also has a large presence in 30 different countries in the African continent, and a smaller presence in the Middle East, Asia and Portugal. Cuba has always offered to help neighbouring countries following natural disasters (Table 1) [4]. For example, in 2010 it played a key role in the provision of health care following the earthquake in Haiti [5]. It also offered assistance to the USA following hurricane Katrina in 2005. The same year, following the earthquake in Pakistan, Cuba sent over 2000 health professionals [6] who stayed longer than any other international group. Cuba was also in the forefront of the recent Ebola outbreak in Africa [7,8] and this latter contribution to global health has won praise from the World Health Organization and its director Dr. Margaret Chan [9].

## 2. Child Health in Cuba

Cuba is a small island with a population of approximately 11 million. It is a middle-income country which plays a major role in the development of the world’s health workforce. How, therefore, has this been achieved? Firstly, it is important to notice that health care, and child health in particular, is excellent in Cuba [5,10,11,12]. Child mortality rates are lower than those of the neighbouring USA (Table 2) [13]. Health outcomes in Cuba are excellent because it offers universal access to free health care. Primary healthcare in particular is well established and focuses among other things on disease prevention [10,11,12]. For example, there is an extensive immunisation programme, which covers 12 different diseases and is universal (Table 3) [10,14,15].

Cuba has one of the highest density of physicians in the world (67.2 per 10,000 population) [16,17]. This has been achieved by ensuring that education is free and accessible by all [10]. Almost half of all Cuban doctors are based in primary health care [11,18]. This means that each family doctor who works in conjunction with a nurse is responsible for approximately 300 families [10,18]. This enables the doctor and/or nurse to see each family member on a routine basis once a year and to be aware of the medical and social problems of each of the individuals within a single family [10,11,18]. This high ratio of doctors to population allows Cuba to send health professionals overseas without it adversely affecting the health of its own population.

## 3. International Health Collaboration

Cuba’s first international health collaboration occurred in 1960 when Cuban doctors were sent to Chile after an earthquake [19]. In 1963, Cuba sent a group of 50 health professionals to Algeria [19]. This marked the start of Cuba’s collaboration with other countries in terms of community capacity building [19]. One of the first major long-term international Cuban health programmes was for the children of Chernobyl (Table 4) [20]. Within days of the nuclear disaster in Chernobyl in 1986, Cuba sent over a medical team. They determined that many of the children would require long-term treatment and arranged for the children and parents to be treated in Cuba. Thus, children with malignancies and neurological diseases received treatment in Cuba and Cuba has continued to offer treatment for these children for over 20 years [20].

In 2004, Cuba, in conjunction with the government in Venezuela, launched a major initiative called Misión Barrio Adentro (Inside the Neighbourhood) [20]. The aim was to provide access to health care for poor Venezuelans who previously had limited access. More than 20,000 Cuban health professionals have been involved at some stage. Over 10,000 primary care clinics have been established with another 800 planned [21]. In 2004 alone, 76 million medical consultations were provided [16] also including dental care. Overall it is estimated that Barrio Adentro has provided health care to two-thirds of the population of Venezuela [20].

Operación Milagro (Operation Miracle) started with the treatment of visually impaired Venezuelans in 2004 [4,20]. Individuals with visual impairment were assessed and, when appropriate, a simple cataract surgery was performed. Initially, the majority of individuals received free eye surgery in Cuba. Subsequently, the programme has been extended to involve other countries, predominantly in Central and South America. To date, over three million individuals have received free eye operations in over 30 countries [2].

More recently, the Cubans have offered assistance through the Pan-American Health Organisation to the Brazilian government. In 2013, the Brazilian government requested health professionals to provide healthcare to the most deprived sections of Brazilian’s society [22,23]. Most of the Brazilian doctors work in the large cities of the most economically advanced regions, the South and Southeast regions [23]. In contrast, in less developed parts of Brazil, 12 million people live in municipalities where not even a single doctor is established [24]. The Mais Medicos (More Doctors) programme has involved over 18,000 health professionals providing health care to an estimated 63 million people in remote rural areas and the outskirts of cities [24]. The programme involves over 5000 Brazilian doctors, over 11,000 Cubans and more than 1500 doctors from other countries in the world. It has ensured that every single Brazilian municipality now has a primary care doctor [24] and resulted in a significant increase in medical consultations in deprived regions of Brazil, mostly for general medical care, diabetes, mental health problems and prenatal care [23]. This has resulted in a significant fall in presentations to the emergency departments of hospitals [23].

## 4. Aims of International Collaboration

A key feature of the work of Cuban health professionals overseas is that, like MSF, they work in the areas of greatest need. It often includes rural areas where the local indigenous population has previously not had any access to health care [19,20]. The aims of international medical collaboration are:

- To reduce morbidity and mortality in the local population.
- To work in conjunction with local health professionals and local health institutions.
- To focus on primary health care and prevention of diseases.
- To provide basic education for the local population (literacy, immunisation, importance of sanitation and hygiene).
- To provide specialist care in hospitals.
- To educate local health professionals.
- To ensure the sustainability of the intervention by training local health professionals and building institutions as appropriate; for example, rural health centers.
- To empower individuals, families and communities to promote and protect health.

Collaborations have been developed following requests from individual countries. In 2008, Cuba formalised its relationship with Pacific Island countries (Solomon Islands, Vanuatu, Kiribati, Tuvalu, Nauru, Tonga and Fiji) [25]. Thirty-three Cuban health professionals were working on the first four of these Pacific Islands. At the same time as sending Cuban health professionals to work in the Pacific Islands, 177 individuals received medical training in Cuba between 2008 and 2010 [25]. The expectation is that these individuals will return to the Pacific Islands and provide healthcare to the Pacific Islanders allowing the Cuban health professionals to then leave.

In 2004, Cuba sent a small group of doctors to Timor-Leste [26] and soon there were 300 Cuban health professionals working in Timor-Leste. At the same time young people from Timor went to Cuba to study medicine. By 2008, almost 700 students were studying in Cuba and another 100 in Timor itself [19,26]. The Cuba–Timor-Leste health collaboration illustrates how Cuba’s approach is different to that of most charities [19,26]. Salvador Allende has identified the interrelationship between health and social determinants as early as the 1930s [19]. Cubans consider that health is a human right and collaborates with other countries to improve the health of the local population because they feel it is their responsibility [3,4,6,19].

## 5. Guatemala

An example of the way Cubans work within a country is Guatemala, where one of the authors worked for three years. There are currently 445 Cuban health professionals working in Guatemala, a country with a population of 14 million [27]. Guatemala has a high under-five mortality rate and significant inequalities [28]. Unlike Cuba, there is a serious issue involving drugs and violence in many areas. Over 40% of the children in Guatemala under the age of five suffer from malnutrition [29]. This rate is estimated to be about 60% within the indigenous communities [29].

Cuban health professionals have been present in Guatemala for the last 17 years. They first arrived following Hurricane Mitch in 1998 [27]. The Cuban contingent consists of doctors, nurses and other health professionals, working in both urban and rural areas. The majority are working in primary health care and are present in over two-thirds of the administrative health areas of Guatemala. Ninety-one health professionals are working in 23 of the 40 hospitals. Seventy-five percent of the medical consultations are in primary health care. There is a strong focus on health care for pregnant women and infants under the age of 12 months. Each year, the Cubans have been involved in more than 100,000 home visits. In hospitals, Cubans have been also practicing more than 15,000 surgical operations each year. Cuba plans to provide assistance for several more years. The Cuban health brigade has been awarded by the Guatemalan government the Orden del Quetzal, the Monja Blanca, and the entire team collectively received the Person of the Year award. These awards illustrate the importance that Guatemalan government gives to the Cuban presence.

## 6. Angola

Cuba has had a long-term relationship with Angola, providing predominantly health professionals and teachers. One of the authors worked in Huila Province, one of the 18 provinces of Angola, for three years. It has a population of over three million inhabitants, high infant and maternal mortality rates, and a high prevalence of communicable diseases including malaria, tuberculosis, acquired immune deficiency syndrome (AIDS) and neglected tropical diseases. One hundred and sixty Cuban professionals worked in Huila Province during 2010 and 2011, and were involved in various sectors of activity shown in Table 5. The majority were health professionals. The second largest group consisted of teachers, teaching at medical schools and universities. Two individuals taught exclusively basic literacy (the Yes I Can programme) at libraries and a variety of other professionals also worked to improve water sanitation. Over a five-year period between 2008 and 2013, the under-five mortality rate in the province fell from 121.5 to 56.3 per 1000 live births. A medical school was established in the province in 2009 with an intake of 60 students. The first of the students graduated in 2014. The majority of teachers in the medical school are currently Cubans but the aim is to make the project sustainable by involving Angolans teachers.

## 7. Volunteering

Cuban health professionals at the time of qualification have the opportunity of volunteering for a Cuban mission overseas and it offers considerable advantages. Firstly, they can gain medical experience about diseases or traumas that are unlikely to be seen in Cuba itself. Secondly, they receive additional financial compensation. Not all Cuban health professionals who volunteer are accepted. An assessment is made of the individual’s attitude towards work, discipline and professional competence [30]. Graduates who have achieved the highest marks are often given the opportunity of working overseas.

Training in the local language is provided before leaving to a non-Spanish-speaking country. Additional training in emergencies is provided where appropriate. Health professional volunteers usually work overseas for a period of two years which includes a two-week holiday where the airfare is paid for them to come back to Cuba. The individual is usually given a choice of two countries. Individuals may choose to work overseas at a set time, but those involved in emergency missions obviously need to be prepared to work overseas at short notice.

## 8. Benefits for Cuba

Initially health professionals were sent overseas in response to natural disasters on purely humanitarian missions. Governments with sufficient economic resources, such as Brazil, China, Qatar and Saudi Arabia, receiving Cuban health workers have paid or exchanged goods (oil in the case of Venezuela) for this support [3]. Poor countries unable to pay for Cuban health professionals have still received assistance. In some cases, high-income countries such as Norway have paid Cuba to send Cuban health professionals rather than sending Norwegian health professionals halfway across the world [3,5].

Providing health professionals is now one of the biggest sources of income for Cuba even if Cuba only receives compensations when the governments can afford to pay. Cuba’s international health collaboration started as a humanitarian response rather than as an economic plan and still the Cubans do not see health provision as a commodity that can be bought and sold but better as an exchange. Cuba provides health professionals to a wealthy country in order to improve the health of the local population (Brazil, China, Qatar and Saudi Arabia all have higher child mortality rates than Cuba). In exchange the wealthy country compensates Cuba. This money can then be used to provide free health care and education in Cuba. Cuba remains committed to universal public health services. There have also been numerous political benefits from sending health professionals overseas especially in neighbouring Latin America countries which may have very different political viewpoints towards the Cuban government. Despite these political differences, all neighbouring countries have now re-established full diplomatic relations with Cuba.

## 9. Latin American School of Medicine

Alongside the Cubans working overseas, Cuba also provides medical training to students all around the world. The Latin American School of Medicine (ELAM) was established in 1999 [31,32,33,34]. The students are usually coming from disadvantaged communities who have been recommended by a community/social organisation. In many cases this is a church or other religious organisation actively involved in the community. The students are funded by scholarships from a wide variety of international organisations [3,31,32,33,34]. To date, over 23,000 students from 83 countries have graduated [3]. Additionally, there are over 19,000 students currently enrolled at ELAM [3]. It is hoped that the vast majority of graduates from ELAM will work as primary care physicians in deprived areas [33]. In 2012, the majority of ELAM graduates from Haiti were actually working in Haiti following graduation [33]. ELAM also accepts American students from under-represented minorities and low-income families [34]. Two-thirds of the American graduates are now working in areas serving deprived communities within the USA [34].

## 10. Solidarity

Solidarity has played a key part in the development of Cuba. As a small island that has experienced an economic blockade by the USA, Cubans are extremely appreciative of solidarity from other individuals, organisations and countries. This has helped foster a very positive attitude towards other individuals and countries that are less fortunate than Cuba [3]. It is worth noting that the first country that Nelson Mandela chose to visit as President of South Africa was Cuba. This was to express his gratitude for the solidarity shown by the Cubans in the fight against apartheid. Cuba will continue to provide health professionals to many disadvantaged communities around the world. Cuban health professionals all feel that the opportunity of working overseas is something that they value. Cuban health professionals appreciate that they have received free education in order to help other people and that this is an integral part of social medicine [19]. Working overseas has now become an integral part of the Cuban health system. Less than 1% of Cuban doctors working overseas become economic migrants [24].

## 11. Can others Emulate Cuba?

Cuba has set an example for other countries. The question remains whether high-income countries which are significantly richer than Cuba can emulate the Cuban model. Many junior doctors in high-income countries would value the opportunity of working overseas in order to (i) gain experience; and (ii) help disadvantaged communities. The concepts of social medicine and social justice are universal and many health professionals around the world feel the same responsibility. At present, junior doctors in Europe who wish to work overseas usually engage in a charity such as MSF. However, junior doctors working for MSF have to leave their training programme to work overseas and are not guaranteed a job on their return. European governments should work in conjunction with organisations such as MSF and fund junior doctors to work overseas. Initially this could be on the basis of one or two individuals within each region, within each country but it would require a significant political commitment from European governments. The UK, for example, could start offering 50 places each year.. The financial investment required would be relatively minor when one considers the huge amounts that governments spend on military expenditure [35]. Cuba has obtained significant financial benefit following its initial political decision to help disadvantaged communities in poor countries. The question remains whether European countries and the European Union are prepared to follow Cuba’s example.

## Figures and Tables

**Table 1:** Examples of emergency assistance by Cuba.

Earthquakes	Chile 1960; Peru 1970; Pakistan 2005; China 2008; Haiti 2010; Nepal 2015; Ecuador 2016
Hurricanes	Honduras 1974; Nicaragua 1988; Guatemala 1998; Haiti 2004; Dominica 2015
Flooding	Nicaragua 1991; Honduras 1999
Tsunami	Indonesia, Sri Lanka 2004
Dengue	Brazil 1991; El Salvador 2000; Honduras 2002
Ebola	Sierra Leone 2014; Liberia, Guinea 2015

**Table 2:** Child mortality rates in Cuba and the USA.

	Cuba	USA
Neonatal	2.3	3.6
Under five year olds	5.5	6.5

Data per 1000 live births for 2015 [13].

**Table 3:** Diseases for which Cuban children are immunized.

Diphtheria
Haemophilus influenza type b
Hepatitis B
Measles
Meningococcus B and C
Mumps
Pertussis
Poliomyelitis
Rubella
Tetanus
Tuberculosis
Typhoid fever

**Table 4:** Examples of international health collaborations.

Programme	Year	Country	Aim	Achievements	Reference
Children of Chernobyl	1990	USSR (Ukraine)	Treatment of children from Chernobyl nuclear disaster	More than 19,000 children have received treatment	[15]
Operación Milagro (Operation Miracle)	2004	Latin America and Caribbean	Free eye surgery, mainly for cataracts	More than three million people have received treatment	[2]
Misión Barrio Adentro (Mission Inside the Neighbourhood)	2004	Venezuela	Providing access to healthcare	76 million medical consultations in 2004 alone	[15]
Mais Medicos	2013	Brazil	Providing access to healthcare	Primary care doctor in every municipality in Brazil	[20]

**Table 5:** Cuban professionals working in Huila Province, Angola, during 2010-2011.

Health		Education		Other Sectors	
Hospitals	63	Medical school	28	Veterinary surgeons	6
Primary health	21	Universities	14	Miners	4
Vector control	10	Basic literacy	2	Electricians	2
Pharmacy	4	Libraries	2	Water engineers	2
Training midwives	3			Forensic medicine	1
Physiotherapy	2				
Total	103	Total	46	Total	15
